# Pneumoperitoneum After a Redo Cardiac Surgery: Patience Can Lead to Victory

**DOI:** 10.7759/cureus.23663

**Published:** 2022-03-30

**Authors:** Alfred Ibrahimi, Selman Dumani, Saimir Kuci, Agron Dogjani

**Affiliations:** 1 Anesthesiology, Mother Theresa University Hospital Center, Tirana, ALB; 2 Cardiovascular Surgery, Mother Theresa University Hospital Center, Tirana, ALB; 3 General Surgery, University of Medicine of Tirana, Tirana, ALB

**Keywords:** prosthetic valve dehiscence, pleural effusion, heart disease, cardiac surgery, pneumoperitoneum

## Abstract

Pneumoperitoneum after cardiac surgery is not usual. It occurs during extended sternotomy, which is sometimes accompanied by the opening of the peritoneal cavity or in cases after previous sternotomies. A 73-year-old man was operated on two times within one month due to prosthetic mitral valve dehiscence. The second operation was accompanied by a lot of complications, including blood loss, respiratory failure, massive pleural effusion, sternal infection, pneumomediastinum, and pneumoperitoneum. The presence of pneumoperitoneum alerted the possibility of intra-abdominal cavitary organ perforation. No surgical approach was chosen because there were no signs of peritonitis. The patient was discharged to home in a good health situation. Sixteen months later, he was recovered at the hospital for heart failure, and after abdominal CT, benign pneumoperitoneum was observed in the abdominal cavity again. There was no explanation for this finding, but again conservative treatment was chosen. He was discharged for the third time from the hospital in good condition.

## Introduction

Pneumoperitoneum is the presence of air in the abdominal cavity. During cardiac surgery and median sternotomy, because of a closed relationship of diaphragm, pericardium, and peritoneal cavity, the sudden opening of the diaphragm may occur, and air can pass freely into the peritoneal cavity. Also, the passage of the drainage tubes at the end of the operation can partially damage the xiphoidal part of the diaphragm creating benign pneumoperitoneum again. The presence of pneumoperitoneum in the early postoperative day doesn't create any concern for medical staff, but in late postoperative days, especially in sedated ventilated patients with complications, it can raise alarm for possible bowel injury in the abdominal cavity. Here we will present a challenging decision-making case regarding pneumoperitoneum in the same patient at two different times. 

## Case presentation

**Figure 1:**
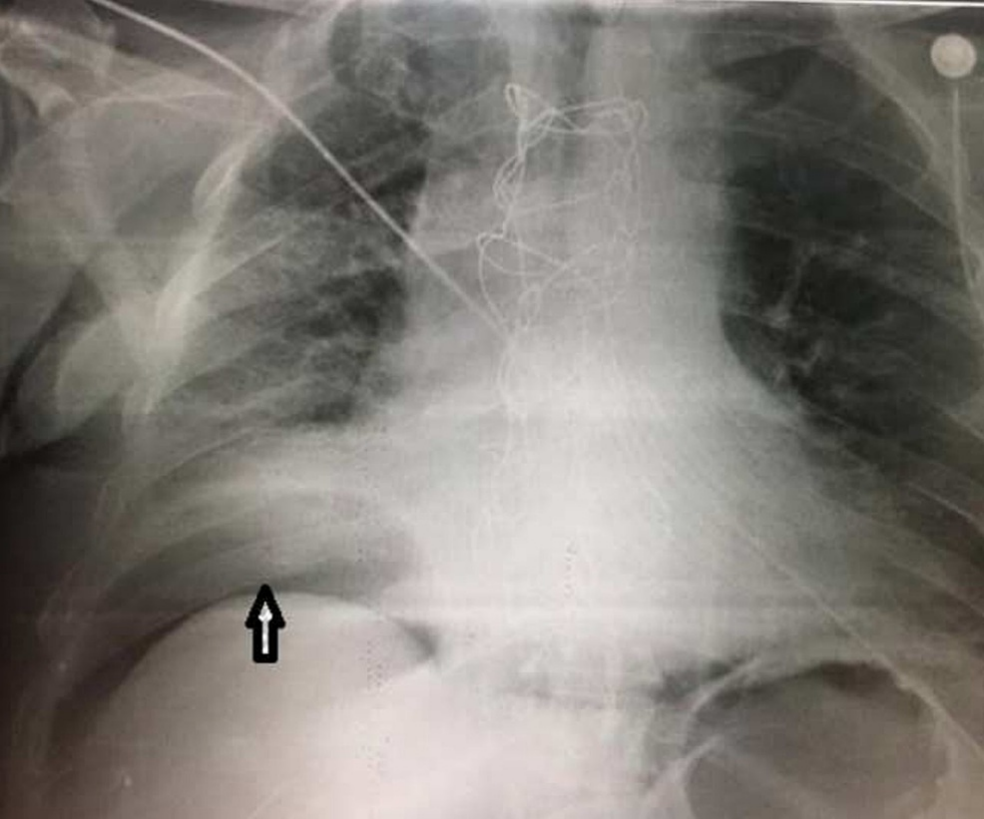
Anterior-posterior X-ray made in the ICU The arrow shows the presence of air between the liver and diaphragm.

A 73-year-old male patient was recovered in our institution suffering from ischemic heart disease and severe mitral valve regurgitation. The patient had a concomitant disease, diabetes mellitus, mild chronic renal failure (creatinine level 1,8 mg/dl), and mild obstructive pulmonary disease. Elective surgery was planned. After median sternotomy and onset of cardiopulmonary bypass (CPB), and after the aortic cross was clamped, cardioplegia was infused in the aortic root. Triple bypass, two with saphenous vein and one with left mammary artery, was performed. The mitral valve was replaced with a prosthetic St Jude number 29 valve. The heart was weaned from cardiopulmonary bypass with the help of small doses of inotropic support. Perioperative and postoperative periods passed without problems, and on the seventh postoperative day, the patient was discharged from the hospital in good health, taking anticoagulation therapy with warfarin, furosemide for heart failure, and supportive therapy for chronic kidney disease. Three weeks later, he represented to our institution complaining about orthopnea, generalized edema, and ascites. On transesophageal echocardiography, severe mitral regurgitation (peri-valvular leak) with prosthetic valve dehiscence was found. After two days of preparation, the patient was sent to the operating room again, with a very high EuroSCORE II predicted mortality score of 62,46%. During the second operation, it was noted that the prosthetic valve number 29 was undersized compared to the annular ring. A second valve, number 33, was implanted again in the mitral position. After the sternal closure, the patient went into cardiac arrest, and after reopening the chest, the patient was placed again on cardiopulmonary bypass. After 30 minutes of CPB support, the patient was weaned from CPB with elevated inotropic support and was sent to ICU. After two days of controlled respiratory assistance and the stabilization of cardiocirculatory and renal parameters, the patient was weaned from respiratory assistance. On the seventh day after the operation, the patient went into severe respiratory distress syndrome, severe polypnea, and severe hypoxia. A chest X-ray and pulmonary ultrasound showed massive bilateral pulmonary effusions. A bilateral chest drain was placed in the midaxillary line. On the first day, 2 liters of clear fluid was drained from the left hemithorax and 1 liter from the right. The drainage was closed for 24 hours and was opened again the next day. Again, 1,5 liters of transudate were drained from the left side and 700 ml from the right. Meanwhile, the wound in its inner part had begun to open because of the infection from where the sternal wires could be seen. Aggressive therapy with antibiotics (imipenem) and wound medication with a low concentration of povidone-iodine was applied. Renal parameters were closely monitored. In a routine chest X-ray examination, the presence of air in the abdominal cavity under the right diaphragm was noticed (Figure 1).

**Figure 2:**
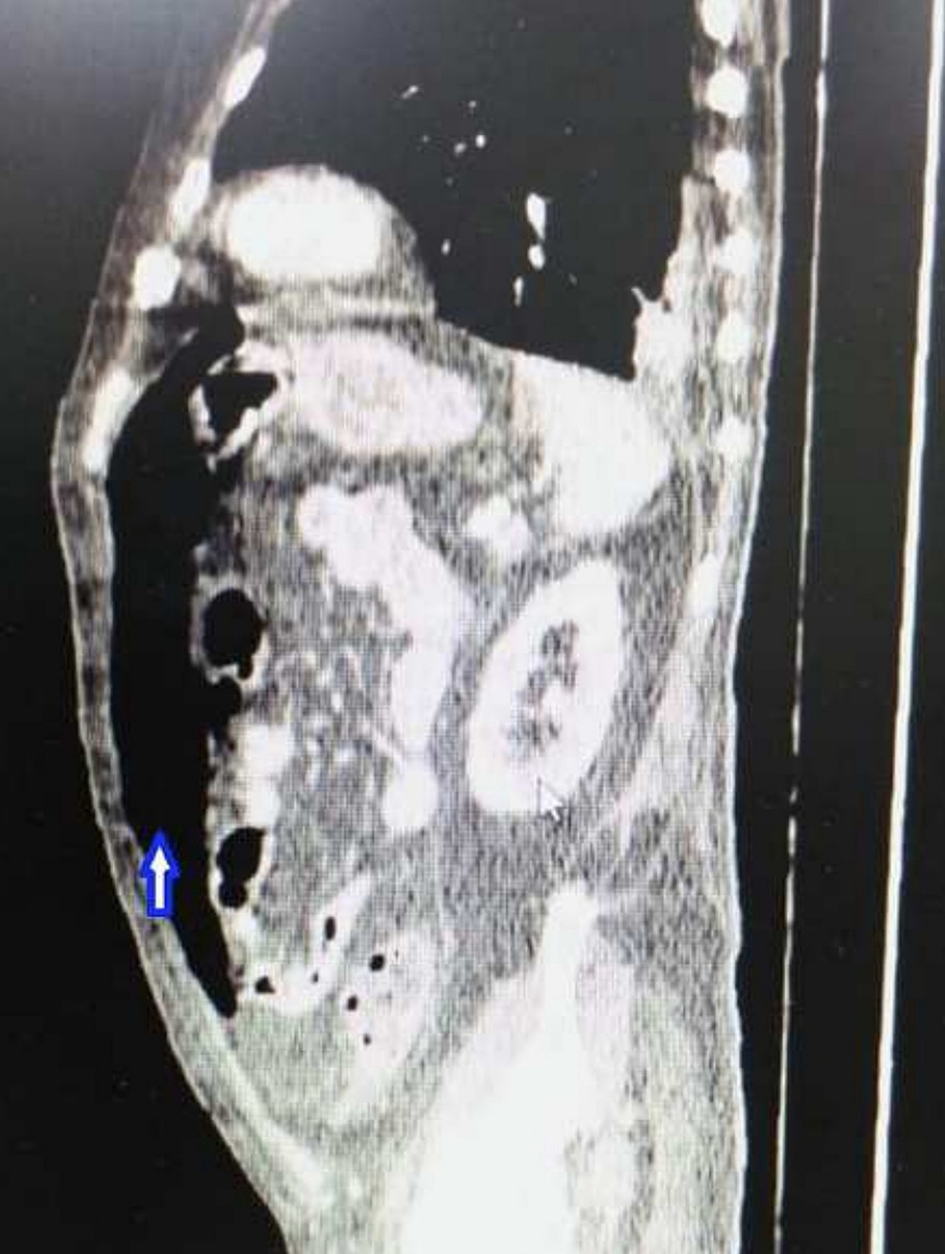
Abdominal CT shows massive pneumoperitoneum in the anterior part of the abdominal cavity

A thoracic abdominal scan was performed immediately where the presence of air was in the anterior mediastinum (pneumomediastinum) and in the abdominal cavity (pneumoperitoneum) (Figure 2). 

The patient didn’t complain of any abdominal disorder or pain, and in auscultation, he had good peristalsis and no signs of acute abdomen. In consultation with the general surgeon, we choose to follow non-operative therapy. For the wound infection, the chest wall was put in a vacuum for seven days, and later again, the patient was sent to the operating room for the reconstruction of the chest wall. The operation consisted of partial mobilization of two major pectoral muscles used as a flap, and closed again with stainless steel because there was no sign anymore of sternal infection. The time of recovery for this third operation was shorter (seven days). The patient was treated for six weeks with antibiotics and supportive therapy. He was discharged from the hospital after two months in good health conditions. Sixteen months later, the patient was readmitted to the hospital, now to the internal medicine, not the cardiac surgery division, complaining of shortness of breath and considerable edema of the lower extremities. Creatinine level was 2,5 mg/dl and blood urea nitrogen (BUN) 80 mg/dl. Left ventricular ejection fraction (EF) was 35%, with the normal function of the mechanical valve in the mitral position. Diuretic therapy of 200 mg furosemide intravenously was started, and the clinical situation was improved again. A repeat abdominal CT scan showed a pneumoperitoneum again. The general surgeon intended to take the patient to the operating room for an exploratory laparotomy but with the information that they got from the cardiac surgical team and the past history that this situation was not new, the decision was made to manage the pneumoperitoneum non-operatively again. After seven days of diuretic therapy, the patient improved by a lot. He was discharged again from the hospital with strict recommendations for routine control from the cardiologist and nephrologist. 

## Discussion

Intraabdominal complications (IAC) occur in 0.5% to 3.0% of all patients undergoing cardiac surgery [1, 2]. They are a significant source of morbidity and mortality. Mortality rates of 14.5% to 100% [2, 3, 4] have been reported from various centers for these complications after open-heart surgery. As a result of perioperative hypoperfusion, IACs appear in several forms. [5, 6, 7]. Clinical manifestations of hypoperfusion and reaction to postoperative stress are a consequence of gastrointestinal bleeding (GI), acute pancreatitis, acalculous cholecystitis, peptic disease, perforated ulcer, nonocclusive intestinal ischemia, diverticulitis, and liver dysfunction.

Of all the above, nonocclusive intestinal ischemia, which then leads to intestinal infarction, is the most deadly of all intra-abdominal complications of hypoperfusion, followed by relative visceral ischemia due to prolonged cardiopulmonary bypass, vasoconstriction, low cardiac output, incorrect perfusion distribution, obstructive splanchnic disease, and embolism [7, 8]. One of the abdominal complications is also a pneumoperitoneum. Pneumoperitoneum is defined as the abnormal presence of air in the abdominal cavity. Pneumoperitoneum has multifactorial etiology originating from the abdomen, thorax, pelvis, but during cardiac surgery, it can be caused by damage to the diaphragm during sternotomy, as well as the placement of thoracic drainage at the end of surgery where the peritoneum can be opened incorrectly, causing communication between the thoracic and peritoneal cavities. 

Pneumoperitoneum may not always require operative treatment because it is not always caused by bowel injury [9, 10, 11]. The presence of air in the abdominal cavity, especially in severely ill patients operated for heart surgery, is a major concern for medical staff because operated patients are sedated in totally opioid analgesia, are sometimes intubated in mechanical ventilation, and in 90% of cases, there is suspicion of one of the complications mentioned above. There are not many published articles about pneumoperitoneum after cardiac surgery. 

The clinical history of the patient is important for the diagnosis. Recent or past events that could cause spontaneous pneumoperitoneum have to be taken into account to avoid unnecessary laparotomy. At the same time, a delay in performing a laparotomy needs to be avoided if there has been a bowel injury. To better understand our case, we should remember what the natural routes of communication between abdominal and thoracic compartments are. Although the idea is controversial, many authors consider aortic and esophageal hiatuses as the most common communication between the thoracic and abdominal cavities [12]. 

The thoracic and abdominal cavities can be seen as a continuation of each other; in fact, in the fetus, they were a single cavity, while in adults, the diaphragm plays an important role as a barrier to air diffusion. But this communication may persist, allowing air to enter the abdominal cavity as well as ascites to leak into the pleural cavity [13].

In our case, initially, we could not explain massive pleural effusion on the seventh postoperative day (4,9 liters, over three days), but after pleural effusion reduced its quantity, pneumomediastinum (no pneumothorax) and pneumoperitoneum were noticed in a routine X-ray. Initially, the abdomen cavity was full of ascites with elevated pressure; the liquid passed through a natural and iatrogenic communication of the diaphragm to the thoracic cavity in pleural space, creating massive pleural effusion. After the number of ascites was reduced, the abdominal pressure decreased, and the air was sucked from the open wound (caused by infection) in the inferior part of the sternum and passed in the same route in the abdominal cavity, creating benign pneumoperitoneum. 

We could not explain the presence of pneumoperitoneum 16 months after the second operation. Usually, the air in the abdominal cavity is absorbed during that time, but we didn't find any sign of abdominal cavitary organ perforation and symptoms of acute abdomen [14, 15]. We had the patience to not make an aggressive decision (surgery), and this way, we prevented an unnecessary laparotomy and saved our patient's life. 

## Conclusions

Pneumoperitoneum is a rare finding during cardiosurgical operation. Based on the experience of our team, we consider prompt exploratory laparotomy, irrespective of the results of the diagnostic tests, as the only way to provide objective assessment and adequate treatment, leading to a dramatic reduction in the mortality rate. It is necessary to understand the origin of air in the abdominal cavity, because laparotomy may not always be indicated to deal with a pneumoperitoneum. Clinical data need to be taken into consideration in addition to the radiological data to make the appropriate treatment decision. 
